# Analyzing the Co-Competition Mechanism of High-Tech Park from the Perspective of Complex Socioeconomic Network

**DOI:** 10.3390/e23080978

**Published:** 2021-07-29

**Authors:** Lizhi Xing, Yu Han, Jingying Xu

**Affiliations:** 1 College of Economics and Management, Beijing University of Technology, Beijing 100124, China; han_yu@emails.bjut.edu.cn (Y.H.); xujingying@bjut.edu.cn (J.X.); 2 International Business School, Beijing Foreign Studies University, Beijing 100089, China

**Keywords:** complex network theory, social network analysis, high-tech enterprises, co-competition relationship, innovation ecological niche

## Abstract

The fusion of “innovation theory” and “ecology” gave birth to a large number of studies on “innovation ecology”, which mainly studies how to build an industrial ecological chain at the regional level, focusing on self-evolution, achieving ecological balance, and enabling the regional economy to take the path of sustainable innovation. This type of research borrows a lot of concepts from ecology and very vividly describes the competition and cooperation relationships formed by various agents in the innovation system, laying a good foundation for qualitative analysis of the inherent dynamics of innovation development. However, many studies focus on the analogous description of ecosystems and economic systems, lacking scientifically and rigorously quantitative empirical research as support. This paper uses network-based indicators such as degree, cluster coefficient, and betweenness centrality to measure the function and position of high-tech enterprises in the Z-Park of a business environment. In this way, we clarify the socioeconomic meaning of the topological structure of the regional innovation system. On this basis, it provides theoretical references for regional innovation development and sustainable development policy formulation.

## 1. Introduction

The natural ecosystem is an organic whole, composed of the biological community, the external environment and the complex relationship between them at a specific spatial range. The interactions and restrictions between communities and their external environment promote the continuous evolution of the whole ecosystem. Competition theory suggests that multiple species are not able to occupy the same niche indefinitely [1], preventing a stable biological coexistence [2]. Closely related organisms of the same niche will intensify the distribution of competition [3], and competition from overlapping niches is not absolute. In community ecology, thus, the biodiversity and coexistence of competing species still remain an open problem [4]. Similar to natural ecosystems, organizational ecosystems are composed of a wide variety of actors and organizations, some of which act as key species in the ecosystems. In the 1970s, scholars introduced the theory of a natural ecosystem to analyze problems within economic organizations. Later, they put forward the concept of an innovative ecosystem, in which technological competition and cooperation bring about technological development and innovation [5] and sophisticated supply chains with multiple players and complex interactions [6].

In recent years, China’s achievements in the industrialization of science, technology, and innovation have attracted international attention. National policies for science parks and innovation have been identified as one of the major driving forces for the economy [7]. The innovation capability of high-tech zones has played a positive role in promoting the innovation capability of the entire country. With the continuous deepening of China’s reform and opening up, it has become an important strategic path to enhance China’s national strength. Touted as China’s Silicon Valley [8], Z-Park covers 250 km^2^ in Beijing, which gives birth to China’s largest cluster of semiconductor, computer, and telecommunication enterprises financed by both domestic and overseas capital [9]. Z-Park generated USD $161.8 billion of industrial output in 2018, registering a year-on-year growth of 3.1%. It is, thus, of great significance to study the innovative co-competitive relationship of enterprises in the Z-Park, considering the geographical agglomeration and policy support. However, at present, many high-tech zones in China are simply gatherings of companies, from a spatial perspective, rather than innovative network structures with complementary functions resulting in insignificant improvements in innovation capability, a lack of growth advantage, and lack of market competitiveness. The innovative network of high-tech zones can greatly facilitate the flow of knowledge and information, integration of complementary resources, and collaborative innovation among enterprises, thus saving costs for innovation activities inside the zone, improving the efficiency of the innovative entity, and enhancing the innovation capability of the entire park.

Therefore, studying the structure and characteristics of the innovation network in high-tech zones and understanding the role of prominent enterprises inside the network bears important theoretical and practical values in guiding the development of technology-based small and micro enterprises inside the network, thereby promoting innovation capability of high-tech zones. Against the backdrop of a knowledge-based economy, product innovation is crucial for the sustainable development of enterprises because innovative products can create new market demands and growth opportunities [10]. Since most synergies between the parkenterprises are commercial transactions and social interactions [11], we established the niche overlap network to depict the overlapping innovation output in niche markets in Z-Park via the network structure description and index analysis. By systematic analysis of the co-competition relation of innovation output, effective suggestions can be put forward on the administration of the science park, thus leading the innovation process and increasing innovation output.

## 2. Literature Review

In 1917, Grinnell proposed the concept of the ecological niche, which he believed referred to the relational position and function of a species in an ecosystem in terms of both time and space [12]. Gause embodied the competitive relationship of an ecological niche and pointed out that if two species in a stable biocoenosis use the same resource simultaneously, one species will have a competitive edge, while the other will be marginalized [13]. The construction of the ecological niche refers to the species’ choice toward the environment. The related theories and empirical studies have gradually become the focal point of evolutionary ecology studies [14]. In the fields of economics and management, the concept of the ecological niche has gradually been acknowledged and applied. The ecological niche of complex regional ecosystems can reflect the fit of various human activities in the region and the advantages and disadvantages of the environment [15]. Innovative entities inside the ecosystem resemble, to some extent, species in the ecosystem, as the subjects also have interspecific relations such as competition and symbiosis. Innovative entities take up ecological niche space in the value chain. That is, the industrial position is based on factors such as innovation resources, functions, and environment. When the ecological niche boundaries of different innovative entities overlap, competitive relations are formed. To some extent, the nature of the corporate relations reflects the interrelationship between enterprise ecological niches [16], i.e., the ability of an enterprise to survive, develop, and compete [17]. Thus, competition among enterprises could be attributed to overlapping ecological niches [18].

The niche breadth of the species indicates the balance between the diversification effect of intraspecies competition and the limiting effect of interspecies competition [19]. The integration of cooperative interspecies interactions in classic niche theory has been considered a major challenge to population ecology and community ecology [20]. Here, the ecological theory is applied to studies of enterprises. The co-competition relationship between enterprises is based on the enterprises’ strategic objectives, which is why local policies can enable the interaction of enterprise competition and cooperation [21]. Systems with cooperation and competition between elements are ubiquitous [22]. In a broad sense, the co-competition relationship between enterprises is defined as the value transfer network formed by the company’s suppliers, customers, competitors, and complementors [23]; in a narrow sense, it is defined as the cooperation between two competing companies [24]. Due to the dynamic business environment, more and more companies are participating in multi-enterprise alliances and developing a series of relationship combinations [25]. Therefore, co-competition relations can exist among more than two enterprises, as many companies can cooperate while competing with each other. In the process of innovation and development, high-tech enterprises have gradually realized that regional-level industrial upgrading is beneficial through survival of the fittest products and services. We believe that the co-competitive game relationship between innovative entities and market demand is the fundamental driving force for shaping the innovation value chain. In competitive clusters, the pure competition that hinders innovative output should be avoided, while in cooperative innovation clusters, cooperative innovation mechanisms should be carefully sought out [26] to create a symbiotic environment and strengthen enterprise cooperation [27].

Following a new trend in empirical research, a large number of studies have explored the co-competitive relationship in economic systems from the perspective of complexity and systems theory. The nature of an innovation ecosystem is the dynamic variation of the enterprises. Along with the evolution of an innovation ecosystem, its network structure will show substantial expansion and path dependency [28], reflecting the traits of Complex Adaptive Systems (CAS) [29]. Therefore, scholars such as Fuller et al. [30] and Gilbert et al. [31] have studied the formation and evolution of innovation networks based on CAS. The complex network, however, is the simplest way to turn the empirical data into models, which are the foundation of analyzing the evolutionary mechanisms at the systems level. For example, the bipartite graph theory is employed to establish a co-competitive network of large and medium-sized companies [32]. Inoue et al. [33] studied the co-competitive network formed by Japanese companies and their patents; Liu et al. studied the development path of high-tech parks in China [34], and Li et al. revealed topological properties of the deterministic and random weighting networks of China’s high-tech industries [35]. Chang et al. showed that the distribution of node degrees complied with the power-law characteristics of general co-competition networks [36]. Meanwhile, Houet al. selected a number of logistic companies to build a complex network competition model and expanded the research scope from monopoly markets to macroeconomic markets [37]. Yang proposed a two-layer complex network model for the analysis of industrial competition relationships and corporate confrontation actions [38]; Gu et al. established a technology innovation network model for the new energy automobile industry and employed social network analysis methods to study network eigenvalues and knowledge flows [39]; and Cheng et al. used complex network analysis methods to analyze network characteristics such as density, point strength, aggregation coefficients, heterogeneity, core edge structure, etc., and the evolution rules of the weighted trade network of international engineering machinery products [40]. Yao et al. established a co-competition model for industrial cluster networks based on the Lotka-Volterra ecological theory and the ecological niche of industrial clusters, thereby exploring the corporate co-competition model and proposing certain coping strategies [41]. Wang et al. studied the impact of internal differences in networks on corporate co-competition strategy selection and performance [42]. Based on social network theory, Wang et al. analyzed the impact of enterprises’ position in the co-competition network of corporate innovation performance through empirical research on the automotive manufacturing industry [43]. Meanwhile, Sun et al. suggested that innovative entities with overlapping niches in high-tech service networks could only avoid niche erosion by adopting a symbiotic cooperation model [44].

In summary, many scholars have constructed a variety of complex network models to describe the co-competition relationship between industrial organizations and employed various network characteristics to explain economic phenomena. However, their research scope mainly focused on industrial organization, which is a microcosm level in industrial economics, and the literature on science park evaluation mainly covers the developed countries of OECD. This paper describes the co-competitive relationships between innovative entities based on the similarity of their outputs and endeavours to establish a complex network model that mirrors the game relationships of innovative entities on the innovation value chain. In an effort to reflect the enterprising ecological niche formed by innovative entities, we review self-organizing mechanisms and reveal the meaning of the economic externalities of the jointly formed enterprise ecological niche.

## 3. Modeling

Through the establishment of complex network models, this paper analyzes the co-competition relationship between high-tech enterprises in the competitive product market. Therefore, we need to figure out the nature of product-based relationships between enterprises and set a reasonable modelling framework.

### 3.1. Modeling Framework

This paper endeavours to construct an Enterprises Niche Markets Overlap Network (ENMON) based on the analysis paradigm of graph theory. The network sets innovative entities in a specific area as nodes, the niche overlapping relationship (that is, providing similar products and services) between them as edges, and then the unweighted and undirected graph $G=\left(V,E\right)$ is formed. The specific construction principles are as follows:

(1) The node set $V=\left\{v_{i}\right\}$ is composed of all the innovative entities within the region, where $i\in \left\{1,2,\cdots ,n\right\}$, and the number of nodes is recorded as $n=\left|V\right|$.

(2) $e_{ij}$ in edge set E represents the business overlapping relationship between innovative entities in the park, which is characterized by the value of $a_{ij}$ in the adjacency matrix $A=\left\{a_{ij}\right\}$. If there is business overlapping between enterprises i and j, then there is an undirected edge $e_{ij}$ between $v_{i}$ and $v_{j}$, expressed as $a_{ij}=1$, otherwise $a_{ij}=0$. As the relationship is bidirectional, $e_{ij}$ and $e_{ji}$ bear the same meaning.

### 3.2. Data Statistics

According to the Z-Park Management Committee’s “Z-Park High-tech Enterprise Directory” (hereinafter referred to as the “Enterprise Directory”), as of November 2018, there were 25,564 high-tech enterprises in the Z-Park. The parks and their technical fields are shown in Table 1 and Table 2 below.

According to the above-mentioned enterprise directory, we collected names of all competitive enterprises in Z-Park through tianyancha.com, which is a business and industrial data and information inquiry system. On tianyancha.com, a column of “competitive product information” can be found under the introductory information of the enterprise one inquires. We collected the competitive product information of Z-Park high-tech enterprises and filtered out the ones that are also located in Z-Park from the selected competitive enterprises. The overlapping business relationship between them can be attributed to the following two reasons: one is the competitive relationship between enterprises due to overlapping niches; the other is the cooperative relationship for win-win purposes. Within the regional economic system, these two types of relationships can be further measured and analyzed through the characteristic indicators of the network topology.

### 3.3. Network Statistics

According to the above-mentioned data and principles, this paper builds a niche overlap network for innovation value chains. Based on the ENMON model, the simplified network ENMON-PA (Park Area, PA) and ENMON-TF (Technology Field, TF) models are constructed by merging enterprise-level data in accordance with park area and technology field, and the sub-network model is extracted from the ENMON model also according to park area and technology field.

The topological structure of the network model plays a determining role in the nature of the whole network and the association of nodes. The diagram below shows the topological structure of the ENMON, ENMON-PA, and ENMON-TF models.

**Figure 1:**
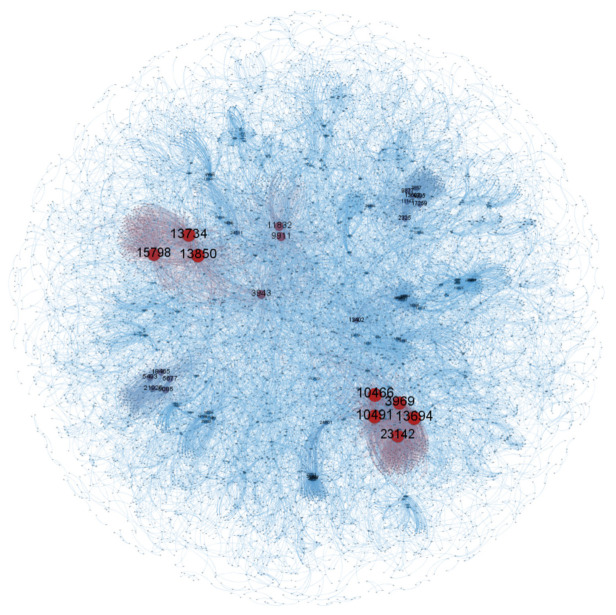
ENMON model. The figure is based on an unweighted and undirected ENMON model. The size and colour of the nodes represent the degree.

As can be seen from Figure 1, nodes in the ENMON model are large in number and densely distributed. The size of most nodes is relatively similar, which shows that the network connectivity of most enterprises is much the same. However, it can be seen, in the figure above, that there are eight companies that are significantly more connected than others and more closely connected to surrounding companies, which are Net 263 Co., Ltd. (10466), Huilan Technology Co., Ltd. (10491), Beijing Tongtech Co., Ltd. (13694), Gongkong (Beijing) Information Technology Co., Ltd. (13850), Business-Intelligence Of Oriental Nations Corporation Ltd. (13734), Beijing Shenzhou Taiyue Intelligent Data Technology Co., Ltd. (3969), Beijing Zhiyun Qidian Technology Co., Ltd. (23142) and Beijing Libiao Xinzhi Technology Co., Ltd. (15798). The reason behind the high connectivity is the characteristics of the electronics and information industry. The niche market of electronics and information products enjoys a high coincidence rate, and with rapid product replacement and no monopoly inside the industry, companies are confronted with fierce competition. In addition, the demand for products is highly flexible and substitutable. Compared with state-owned enterprises, private enterprises have greater customer mobility, which has also intensified competition among companies. Therefore, in order to stabilize the competitive advantage and expand the customer base, companies must increase investments in research and development and constantly innovate to increase their competitiveness in the industry.

**Figure 2:**
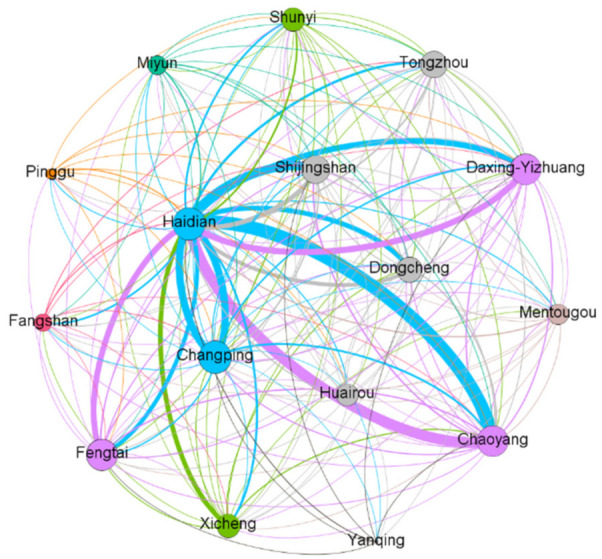
ENMON-PA model. The size and the colour of the node represent the degree, and the thickness of the edge represents the edge weight. The colour of the edge is consistent with the source node.

**Figure 3:**
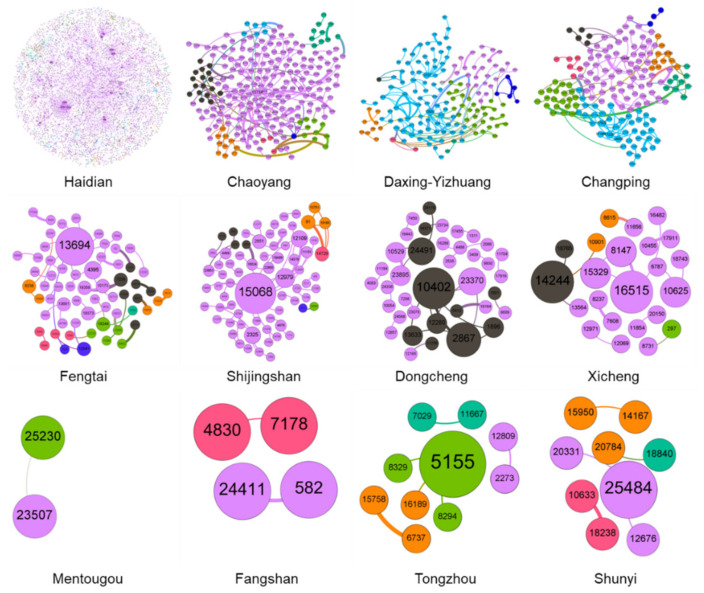
ENMON model sub-network based on park region. The size of the node represents the degree, and the thickness of the edge represents the edge weight. The colour of node and edge represents module partition.

It can be seen from Figure 2 that among the 16 sub-parks in Z-Park, Haidian Park has the closest connection with other parks, which indicates that the competition between Haidian Park and other parks is the most intense and that the overall innovation capability of Haidian Park is higher than that of other parks. From the perspective of enterprises, the four Internet companies, namely Huilan Technology Co., Ltd. (10491), Gongkong (Beijing) Information Technology Co., Ltd. (13850), Beijing Zhiyun Qidian Technology Co., Ltd. (23142), and Beijing Baidu Network Technology Co., Ltd. (3943) have greatly promoted the innovation capability of Haidian Park. Comparing Figure 2 with Figure 3, Haidian Park has the highest density, reflecting that it is the strongest among all parks in terms of collaborative innovation. This is consistent with the fact that Haidian Park has the biggest number of competitive enterprises.

**Figure 4:**
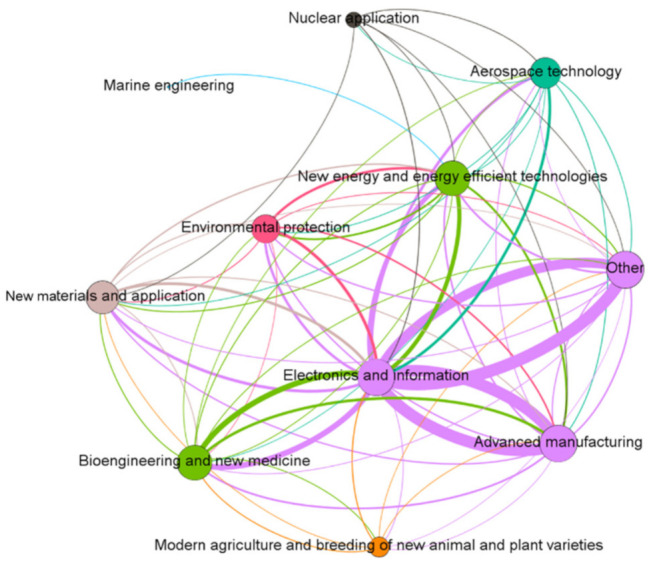
ENMON-TF model. The size and the colour of the node represent the degree, and the thickness of the edge represents the edge weight. The colour of the edge is consistent with the source node.

It can be seen from Figure 4 that among the ten high-tech industries, the edge connected to the electronics and information industries is significantly thicker than that of the others. The electronics and information industries are the most connected, which indicates that in the competitive network, the electronics and information industries enjoy the strongest innovation capability due to competition. This precision is because of the ongoing integration of the new generation of information technology in the manufacturing industry, resulting in higher connectivity between the electronics, information, and high-tech industries. The development of major technology fields requires electronics and information industry-related products. On this basis, in addition to the cooperation between various industries and the electronics and information industries, competition is gradually formed. This type of competition between industries has enhanced the innovation capability of the electronics and information industries. At the same time, the number and scale of companies in the electronics and information industries are also growing.

**Figure 5:**
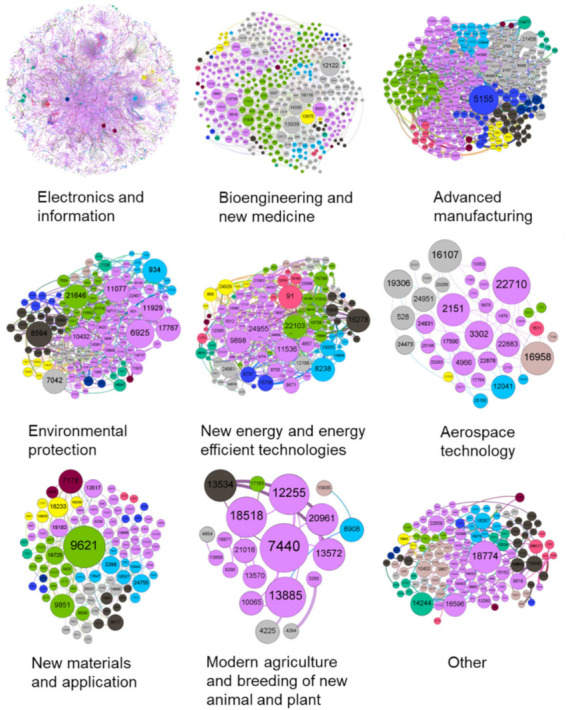
Sub-network of ENMON models based on technical fields. The size of the node represents the degree, and the thickness of the edge represents the edge weight. The colour of node and edge represents module partition.

It can be seen from Figure 5 that the number of enterprises in the electronics and information field is the largest and the competition density is the strongest. As a result, competition between companies in the electronics and information field has promoted the improvement of the innovation capability of the industry (According to Porter’s Five Forces model, enterprises in a fierce competitive environment will face more existing or potential competitors and possible substitutes. At the same time, the stronger bargaining power of upper and lower enterprises will also increase the cost of enterprises. Thus, if an enterprise wants to survive, it must create new profit growth points through business model innovation or technological innovation. Therefore, the high-intensity competitive environment will become one of the sources of enterprise innovation motivation). In the ten major fields, the competition network density in the two fields of aerospace technology and modern agriculture and breeding of new animal and plant varieties is small, reflecting the weak competition-driven innovation behaviors in these two fields. Therefore, promoting the development of the electronics and information industries can facilitate the development of various high-tech areas through cooperation and also prompt other areas to carry out technological innovation by utilizing the role of competition networks. At the same time, strong policy guidance in other fields should be implemented to stimulate the vitality of the industry through competition mechanisms, thereby improving innovation capability.

## 4. Measurement

### 4.1. Degree

Degree *K*, also referred to as connectivity, is the simplest yet most important concept to describe and measure the property of a node. The degree of a node is the number of edges that connect it to the node *v_i_*. The degree of a node is positively correlated with its importance in the network [45]. It can be defined based on its adjacency matrix:

(1) $$K\left(i\right)=\underset{j\in \tau \left(i\right)}{\sum }a_{ij}$$

In social networks, the degree can be used to indicate the influence of individuals. The higher the degree, the greater the role of the individual in an organization. Therefore, under the ENMON model, a higher node degree indicates stronger competition intensity faced by the innovative entity due to its ecological niche. In addition, the degree distribution reflects the topological structure of the innovation ecosystem and can be readily utilized to carry out horizontal analysis when comparing different systems. The characteristics of the ENMON network shows that companies with higher degrees will face a fiercer product market. The market mechanism of “survival of the fittest” puts higher requirements on the development of such companies. The overlap of their niche requires greater efforts of innovation in order to occupy a larger space; otherwise, the company will be eliminated by the market.

### 4.2. Cluster Coefficient

The clustering coefficient, *C*, is a measure of the degree to which nodes in a network tend to cluster together, that is, the degree of familiarity between the nodes. In a Boolean network, the clustering coefficient of a node describes the connection relationship between the node and other directly connected nodes in its network. The quantitative value is equal to the ratio of the actual number of edges that exist between these adjacent nodes to the maximum possible number of edges. The formula is:

(2) $$C\left(i\right)=\frac{A\left(i\right)}{\frac{1}{2}K\left(i\right)\left(K\left(i\right)-1\right)}=\frac{2A\left(i\right)}{K\left(i\right)\left(K\left(i\right)-1\right)}$$

In the above formula, $A\left(i\right)$ is the actual number of edges between the node $v_{i}$ and its adjacent nodes. If the node $v_{i}$ has one or zero adjacent nodes (that is, $K\left(i\right)=1$ or $K\left(i\right)=0$), then the numerator and denominator of the formula are both 0, or $C\left(i\right)=0$. In the ENMON model, the clustering coefficient can be used to describe the intensity of competition among enterprises under the state of industrial clusters, which is called competition density. The intensive competition also brings a lot of opportunities for cooperation. The enterprises with higher innovation density in the ENMON network are also braced with potential cooperation opportunities. When competing with their products, companies also draw on the strengths of rival companies, thus realizing common development under the co-evolution mechanism.

### 4.3. Betweenness Centrality

The Structural Hole theory proposed by American sociologist Burt shows that a lack of direct contact or ties between two or more entities creates holes in the network. Burt believes that based on his structural hole theory, that individuals with high betweenness have information and control advantages, which can be utilized to control other individuals connected to them and obtain intermediary benefits [46]. Freeman proposed a method for measuring structural holes—betweenness centrality $C_{B}$, or betweenness for short, which describes the conduction effect of nodes in the network [47]. If there are $d_{jk}$ shortest paths between a pair of nodes, and $d_{jk}\left(i\right)$ of them passes through the node $v_{i}$, then the contribution rate of the node $v_{i}$ to the betweenness of the pair of nodes is $d_{jk}\left(i\right)/d_{jk}$. The betweenness $C_{B}\left(i\right)$ of node $v_{i}$ may be normalized by dividing the sum of the contribution rates of the node $v_{i}$ to all nodes in the network by the total number of node pairs. The formula is as follow:

(3) $$C_{B}\left(i\right)=\underset{i,j,k\in \left\{1,2,\cdots ,N\right\}}{\sum }\frac{d_{jk}\left(i\right)}{d_{jk}}\left(i\neq j,i\neq k,j\neq k\right)$$

Betweenness centrality measures, in a scientific way, the transitive effect of a node or an edge on the flow of information between other nodes. Therefore, in empirical research, this index is usually used to quantifying the potential control of Hub nodes or edges to the information flow. For the ENMON model, the betweenness centrality can effectively identify which innovative entities are important in the innovation network formed by the innovation value chain. Companies with high betweenness centrality are affected by the merger and reorganization mechanism. They usually merge upstream and downstream companies or achieve cross-industry mergers at the intersection of the value chain, integrating different industry and product content on various industry chains to promote comprehensive.

### 4.4. Efficiency

Latora [48] first proposed the concept of “efficiency” and applied it to the study of efficiency behaviours in small-world networks. Efficiency can measure the effectiveness of information exchange between network node pairs. The global efficiency of a complex network can reflect the effectiveness of information dissemination.

The network efficiency is evolved from the average path length L. Previous studies used the L to measure the reliability of complex networks (the larger L, the lower the network reliability), but these studies are found to be defective. With the average path length index L being improved, the network efficiency becomes a signifier of the network reliability. The reciprocal of the shortest path length between node i and node j refers to the efficiency between the two nodes, i.e., $\varepsilon_{ij}=1/d_{ij}$, where $d_{ij}$ represents the shortest path length between node i and node j. If the two nodes are not connected, $d_{ij}\to \infty ,\;\varepsilon_{ij}\to 0$. The global efficiency of the network is the average value of the efficiency between all node pairs, denoted as E and formulated as:

(4) $$E=\frac{1}{N\left(N-1\right)}\underset{i\neq j}{\sum }1/d_{ij}$$

where 0≤E≤1. When E=0, it means that there are only isolated nodes in the network without connecting edges between nodes; when E=1, it means that all the node pairs in the network are directly connected. The greater the network efficiency, the better the connectivity between the nodes and the stronger the network reliability.

For the overlapping network of an enterprise niche market, the global efficiency E is an indicator of a network’s co-competition pattern, reflecting the ability of resources and information dissemination in the network through competition and cooperation. The higher the network efficiency, the closer the overlap of the main product niche market, meaning the enterprises are facing more extensive competition but also more channels for cooperation.

## 5. Results

### 5.1. Innovation Intensity

According to the statistical results in Table 3, the 20 companies with the highest node degrees in the ENMON network all fall into the field of electronics and information. Compared with other high-tech industries, electronics and information products have a wide range of applications and varieties and can be easily replaced. They’re upgraded and changed frequently, with short life cycles and complex version control. Due to the higher demand elasticity and novelty, the electronics and information industry barely gives rise to a monopoly economy, and the industry competition is becoming increasingly fierce with technology research and development.

The electronics and information industry has its unique competitive characteristics. The traditional economy is driven by economies of scale, but the driving force of electronics and information is mainly the network economy. In this industry, the value of the company to a user depends on the number of users its products have. Therefore, the network’s property of “connection with the best” results in the positive feedback of “the stronger get stronger, and the weaker get weaker”. It can be inferred that the degree of connectivity of the enterprise cooperation network in the electronics and information field is also stronger than that of other types of industries. Under the competitive mechanism of survival of the fittest, companies with greater innovation breadth face abundant competitors in the market. Thus, they have to enhance their innovation capabilities, or they will be eliminated by the market, a testament to the role of the market in resource allocation. Competition is also a catalyst for cooperation. The competition in the electronics and information field has also promoted opportunities for cooperation between enterprises. Hence, it’s possible that the connectivity of the enterprise cooperation network in the electronics and information field is also stronger than that of other types of industries.

In addition, among the top 20 companies, only China Software & Technology Services Co., Ltd. (18465), Beijing Guoxin Tianchen Information Security Technology Co., Ltd. (5085), and ChinaSoft Information System Engineering Co., Ltd. (21925) are state-owned enterprises, ranking 12th, 13th, and 16th respectively. A safe conclusion can be drawn that in the innovation ecosystem, private enterprises have greater innovation vigour than state-owned enterprises.

### 5.2. Innovation Density

From the clustering coefficient under the ENMON model, it can be found that among the 8464 innovative entities, the clustering coefficient of 33.34% of the innovative entities is 1, and 54.57% of them have a cluster coefficient of 0.5 and above. In the 100 null models generated by random reconnection, the probability of the same situation is almost 0, and the network average cluster coefficient is only 0.0017, indicating that the distribution of cluster coefficient in the ENMON model is far from randomly formed and high-tech enterprises in the innovation ecosystem have high competition density. This is related to the concentration of the high-tech enterprises in Z-Park. In Z-Park, the majority of high-tech enterprises belong to the electronics and information technology field. With the rise of “Internet +” (“Internet +” (i.e., “Internet Plus”) means Internet plus various traditional industries. This is not a simple addition of the two, but based on information technology and the internet platform, the internet and traditional industries will be deeply integrated with the advantage of the internet, creating new development opportunities) in recent years, a large number of companies have emerged, leading to fierce competition. The services and products provided by these companies are very similar, which breeds competition. The high competition density has stimulated the development of high-tech enterprises and has formed a good catch-up trend in the high-tech park. Turning competition into a driving force for development and cooperation is an important direction for the development of high-tech parks. To do so, enterprises need to conduct independent R&D, mergers and reorganizations, or cooperative development. Still, fierce competition also requires the government to manage these enterprises in an effort to prevent vicious competition.

### 5.3. Innovation Depth

Table 4 lists 20 high-tech enterprises with the highest betweenness centrality under the ENMON model. Here, the betweenness centrality reflects the key role of the innovative entities in the innovation ecosystem. As can be seen from the table, among the top 20 companies, electronics and information fields account for a large proportion, which indicates that these companies are the main intermediaries in the competition network. Among them, Beijing Baidu Network Technology Co., Ltd. (3943) has a betweenness centrality much higher than other companies, and therefore plays the most important intermediary role in the network. The company is a subsidiary of Baidu, with multiple business lines such as web search, hao123, and Baidu advertising. As the world’s largest Chinese search engine, it covers a wide range of fields and has played a platform role in the competition among enterprises. At the same time, companies involved in the cloud computing business, like Beijing Baidu Network Technology Co., Ltd. (3943) and Beijing Qihoo Technology Co., Ltd. (12971), and those with big data business, like Beijing Baidu Network Technology Co., Ltd. (3943) and Huilan Technology Co., Ltd. (10491) also play an intermediary role in the innovation network for their advantages in extensive information.

For Haidian Park, large enterprises play a better intermediary role in the network than other parks. Among them, Beijing Baidu Network Technology Co., Ltd. (3943), Beijing Sina Internet Information Services Co., Ltd. (10479), Gongkong (Beijing) Information Technology Co., Ltd. (13850), Economic Century Medical Network Technology (Beijing) Co., Ltd. (11352), Huilan Technology Co., Ltd. (10491), and Beijing iQIYI Science and Technology Co., Ltd. (13402) are the top six companies with the highest betweenness centrality in Z-Park. This means that there are many innovative entities in Haidian Park, and they have promoted the development of the innovation network to a certain extent.

However, if the betweenness centrality of an individual is too high, or the individuals differ a lot from each other, some defects may arise for the network as a whole. This means that the broker occupies too high a position, and the connections between individuals are uneven. Specifically, such a network is very vulnerable. The problems with a single critical node can have a great negative impact on the entire network. Furthermore, the heterogeneity of the network is obvious, and resources tend to go to large enterprises, resulting in the phenomenon of “the stronger get stronger, and the weaker get weaker”. This will inevitably hinder the development of small and micro-enterprises.

Table 3 and Table 4 show that there is a total of six companies that are ranked among the top 20 in both tables. They are all companies in the electronics and information field: Huilan Technology Co., Ltd. (10491), Gongkong (Beijing) Information Technology Co., Ltd. (13850), Business-Intelligence of Oriental Nations Co., Ltd. (13734), Beijing Zhiyun Qidian Technology Co., Ltd. (23142), Beijing Baidu Network Technology Co., Ltd. (3943), and Beijing iQIYI Science and Technology Co., Ltd. (13402). In recent years, Internet companies have been increasing R&D investment and supporting the development of the physical industry. At the same time, they have actively promoted the large-scale commercialization of the industrial internet platform and the digital, networked, and intelligentized development of the manufacturing industry. They have provided robust support for the new model and new business forms of intelligent production, collaborative manufacturing, personalized customization, and service extension. All these efforts have helped improve the quality and efficiency of the real economy. Therefore, these companies play a vital role in the innovation network. In order to promote the development of the innovation ecosystem, we should continue to enhance the innovation and R&D capabilities of internet companies, to fully utilize their role as platforms and intermediaries.

### 5.4. Topological Analysis

The global efficiency E is an indicator of a network’s co-competition pattern, reflecting the ability of resource and information dissemination in the network through competition and cooperation. The higher the global network efficiency, the closer the connection between nodes in the network, indicating that a relatively mature co-competition pattern of products has been formed and that information and technological innovation are more efficiently spread and disseminated. The following figure shows the distribution of network efficiency in Z-Park’s sixteen parks and eleven technical fields and depicts the product co-competition pattern of Z-Park Science Park in different regions and industries.

**Figure 6:**
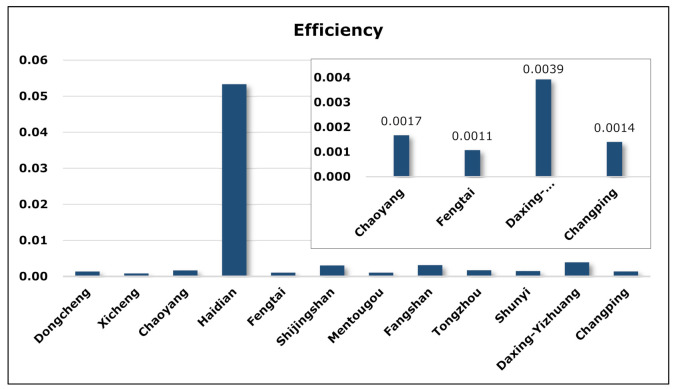
Network efficiency of parks in ENMON model.

As seen from Figure 6, the network efficiency of Haidian Park ranks first among the 16 parks of Z-Park, far exceeding others, among which Daxing-Yizhuang Park has high network efficiency. As one of the earliest established parks, Haidian Park has accumulated a large number of high-tech enterprises thanks to the strong innovation capacity brought by universities and research institutes and years of related experience, with massive innovation output and severely overlapped value chains. On the other hand, although the competition for innovative products is fierce in Haidian Park, major leading enterprises, including Tencent and Xiaomi, grow larger and lead the market competition, which has led to a highly efficient product competition network. Furthermore, the network has not only improved the innovative information flow and transformation capabilities formed by enterprises in product competition but also strengthened the “invisible cooperation” between enterprises in terms of technology.

**Figure 7:**
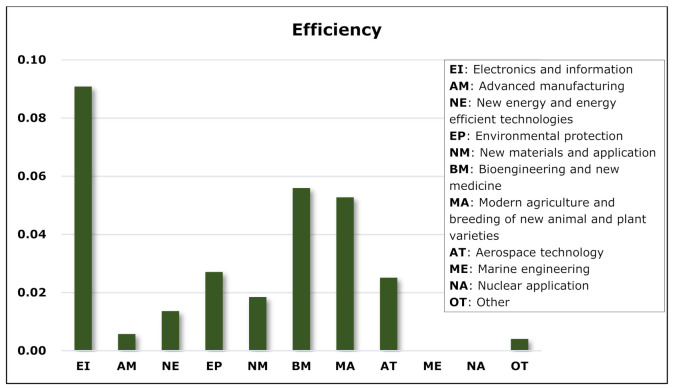
Network efficiency of technical fields in ENMON model.

It can be clearly seen from Figure 7 that the network efficiency in the electronics and information field is higher than in other technical fields. After a long period of development, the electronics information field boasts a large number of enterprises and a smooth supply chain and value chain. However, due to the rapid upgrading rate of electronics information products, some enterprises tend to imitate trendy products, which is unfavourable for product innovation and the development of the entire industry. In order to give full play to the innovation of enterprises in the electronics information field and reduce waste of resources caused by disorderly and malicious competition, division of labour or technical cooperation should be formed under the existing competition chain, respective product markets should be segmented, and overlap of ecological niche reduced. The modern agricultural innovative products are found to be notably homogenous. The reason is that the main products are mostly from feed production and livestock breeding and that the market is mainly concentrated in advantageous enterprises.

**Figure 8:**
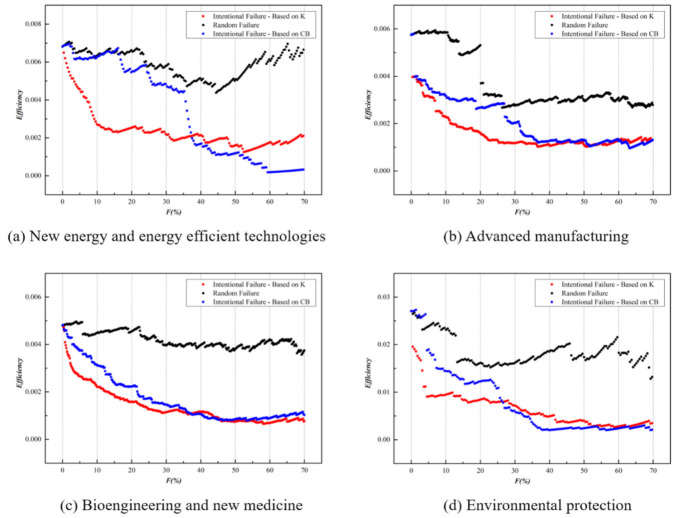
Cascading failure of four main technical fields in ENMON model.

Our research is focused on the enterprises and applies three attack strategies: random attack, K-based intentional attack, and $C_{B}$-based intentional attack, so as to examine the impact of core enterprises on the global efficiency of the network in different parks and technical fields. The following section describes a simulation of the ENMON network reliability and thus analyzes the changes in network efficiency under different attack strategies. In order to fully reflect the actual situation and underline comparison, four parks and technology fields are selected for simulation, considering their large number of enterprises. (The Haidian Park and the electronics information field are excluded for they have too many enterprises for simulation.) The variation trend of the global efficiency in different parks and technical fields is shown in the following figure:

According to Figure 8, under the three attack strategies, as the failure rate of the entities increases, the global efficiency *E* of the competition networks in the four technical fields all display a downward trend, and under the K-based intentional attack, the network efficiency decreased more sharply. It can be noticed that, instead of companies with leading $C_{B}$, those with a larger degree are actually playing the role of the central node in the network and have a greater impact on the network efficiency. High betweenness centrality in the niche overlap network indicates that the main products are related to different competitive groups, which expands the opportunity for the entity to have interregional and cross-domain innovation cooperation. However, in the existing networks of Z-Park, most companies focus only on their own fields when launching innovative products and lack sufficient knowledge and channels for technical integration and product cooperation with other fields. Especially in the NE field, the degree K has a significant impact on the network. When the failure rate is 8%, the K-based intentional attack causes about a 50% decrease of the global efficiency *E*. Under the $C_{B}$-based intentional attack, when the failure rate reaches 36%, the network efficiency drops 50%. The NE field is yet to have large-scale product competition on account of the scattered and unconcentrated manufacturers. Some nodes in the network are indeed highly competitive, but few can actually promote product cooperation, and there are insufficient channels for resource sharing, technology circulation, and scale management. The same situation also occurs in the field of environmental protection. Under K-based intentional attacks, the global network efficiency drops by 50% when the failure rate is about 3%, while under $C_{B}$-based intentional attacks, the network efficiency drops by 4%. In this field, upstream and downstream of the industry chain overlap to some extent, such as steel, electricity, chemistry, and other industries, so the demand side may also turn into the supply side. Focusing only on specific products is not adequate for industrial growth. Further integrating the value innovation chains and promoting cooperation with high-tech enterprises of other fields are the top priorities for the sustainable development of the environmental protection industry. In the advanced manufacturing field, K-based intentional attacks cause a significant decrease in network efficiency. A failure rate of 7% leads to a 50% efficiency reduction, and when the failure rate is less than 5%, the two attack strategies exert influence on the network efficiency without significant difference. Product cooperation between advanced manufacturing and other technical fields is relatively commonly seen. In the field of bioengineering and new medicine, under the K-based intentional attack, a 50% efficiency drop corresponds to a failure rate of 9%. This field has weaker heterogeneity, compared with others, which can be ascribed to the high product differentiation and a large number of drug types.

**Figure 9:**
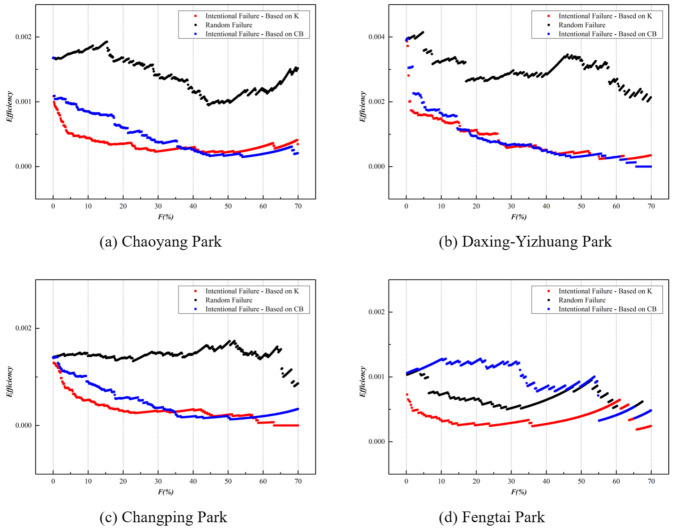
Cascading failure of four main parks in ENMON model.

Similarly, as shown in Figure 9, the network global efficiency *E* of the four parks also shows obvious heterogeneity. The degree K plays a prominent role in the overall network efficiency, which is notably displayed in the Daxing-Yizhuang Park. When the failure rate is 1.15%, the *K*-based intentional attack reduces the network efficiency by 50%. However, as can be seen from the above figure, the two attacks exert almost the same influence on network efficiency. As the core area of the national bio-industry base and the national new industrialization demonstration base, Daxing Bio-medicine Industry Park has gathered a large number of core enterprises in the bio-medicine field, which increases the heterogeneity of the network, and the geographical concentration and government support also expand the channels for product cooperation in the park, leading to the increasing influence of CB on the network efficiency. In the network of Chaoyang Park, when the failure rate is 1%, the *K*-based intentional attack causes the network efficiency to drop by 47%, and the $C_{B}$-based intentional attack causes the drop by 38%. Most of the important nodes that affect network efficiency are electronics and information companies such as Beijing Orient Ntl Comn Sc & Tch Co Ltd. Furthermore, in Changping Park, there are large enterprises in the electronics and information fields such as Net263 Co., Ltd., China National Software & Service Co., Ltd. When the network failure rate is 5%, the *K*-based intentional attacks reduce the network efficiency by 48%, and the $C_{B}$-based intentional attacks reduce efficiency by 23%. There is a high degree of overlap in product markets in the electronics information fields, and large enterprises in the field usually have a significant impact on regional competition. When the failure rate reaches 45%, the network efficiency of Fengtai Park shows an upward trend, which is due to the number of nodes in the network declining faster than the internodes efficiency when the attack reaches a certain percentage, so the network efficiency appears to have an upward trend.

## 6. Discussion and Conclusions

The competition and cooperation between enterprises are not absolute or dynamic. With the cluster’s overall competitive advantage and the need for their own development, enterprises in the cluster are in constant competition and cooperation, forming new co-competition relationships. The niche overlap will cause enterprises to face the pressure of development in the early stage of innovative products and compete for limited resources. However, the ever-expanding competition, based on product integration and technological upgrading, will prompt enterprises to seek cooperation for more competitive advantages. In the past, most of the studies on co-competition enterprises used qualitative analysis combined with game theory to explain the motivation, performance, and development trends. There are also studies with enterprise data being analyzed, but separately studying enterprise technical cooperation or market competition. In order to make up for the deficiencies of existing research in quantifying the co-competition relationship between enterprises and in depicting the status of co-competition between enterprises in the cluster, this paper uses complex network theory methods to quantify the co-competition between enterprises based on the true innovative product competition relationship and examines the co-competition status among high-tech enterprises in Z-Park from multiple angles.

There is fierce enterprise product competition going on in Z-Park, and the product similarity is relatively high. The above analysis has proven that product competition among enterprises has a positive impact on enterprise innovation. First, among the ten major technology fields, the electronics and information industries play a fundamental role in improving the innovation capabilities of major technology fields by competing with other industries. Secondly, given its special industrial characteristics, the electronics and information industries experience fierce competition, with their innovation capabilities ranked first among the top ten fields. Their unique competitive characteristics can also lead to the phenomenon of “the stronger get stronger, and the weaker get weaker”. In addition, regarding the competition network, since private enterprises do not enjoy a relatively stable customer base, their competition intensity is greater than that of state-owned enterprises, which has led to a greater innovation breadth for private enterprises than state-owned enterprises. Finally, the formation of a competition network is inseparable from the contribution of internet companies. From the perspective of the transmission efficiency of innovation elements, internet companies have played an extremely important role in the entire competitive network.

From the perspective of enterprise product cooperation, through cascading failure analysis, the current product competition between the main parks of Z-Park and the technology fields is found to be relatively one-sided. Innovation competition intensity indicators have a greater impact on the overall efficiency of the network than the innovation competition depth. Enterprises with higher competition intensity in the network have a stronger influence on the efficiency of innovation and competition. This implies that most enterprises are still focused on the competition for niche markets and the expansion of their market share, instead of enhancing their advantages through inter-enterprise product and technology cooperation, especially cooperation across technology domains, to occupy market segments. In order to further develop the innovation network and form a sounder innovation ecosystem, it is necessary to give further play to the fundamental role of the electronics and information industries and strengthen the transmission role of internet companies. Meanwhile, relevant policies are required to expand the scope and density of competitive networks in each park to promote the innovation capacity of the park. The specific suggestions are as follows: first, addressing the overlapping ecological niches should be prioritized to promote the complementary development of enterprises. Second, the park should encourage fundamental R&D and closely watch the development of Internet companies. They should also encourage internet companies to continue to innovate by providing them with reasonable support to avoid severe problems that may wreak havoc on the development of the entire high-tech industry in the park. Third, measures should be taken to promote the inter-enterprise integration and cooperation of innovative products, especially cooperation across technological domains. Channels should be opened for enterprises to communicate innovative ideas and share resources. Highly technical innovative products with realistic values will be launched, facilitating the transformation of pure product competition for win-win cooperation.

There are inevitably some limitations in our analyses. In addition to the competition among enterprises, the innovative product relations of high-tech enterprises also include the cooperation between different enterprises based on the upstream and downstream supply chains. Furthermore, the technological upgrading and cooperation that permeate the competition of enterprise innovation products are also worthy of research in the enterprise innovation network. Because of the data limitation, our analysis focuses mainly on product competition, with inter-enterprise cooperation and innovative product upgrading being excluded, the gap of which is thus needed to be filled in future studies. In addition to the product interaction between innovative entities, the market’s response affects the innovation output and competition of high-tech enterprises, which are indispensable but easily ignored. Future research should take these elements into consideration.

## Figures and Tables

**Table 1:** Distribution of High-tech Enterprises in the Z-Park.

Park	Number of Enterprises	Park	Number of Enterprises
Dongcheng	510	Tongzhou	414
Xicheng	738	Shunyi	365
Chaoyang	1749	Daxing-Yizhuang	1378
Haidian	12,939	Changping	3547
Fengtai	1982	Pinggu	134
Shijingshan	933	Huairou	163
Mentougou	183	Miyun	150
Fangshan	262	Yanqing	112

Data source: Z-Park Science Park Management Committee.

**Table 2:** Technical Fields of High-tech Enterprises in the Z-Park.

Technical Field	Number of Enterprises
Electronics and information	15,806
Advanced manufacturing	2227
New energy and energy-efficient technologies	1453
Environmental protection	1082
New materials and application	1181
Bioengineering and new medicine	1776
Modern agriculture and breeding of new animal and plant varieties	287
Aerospace technology	213
Marine engineering	12
Nuclear application	36
Other	1491

Data source: Z-Park Science Park Management Committee.

**Table 3:** The 20 high-tech companies with the highest node degrees in the ENMON model.

Ranking	Company No.	Company Name
1	10466	Net 263 Co., Ltd.
2	10491	Huilan Technology Co., Ltd.
3	13694	Beijing Tongtech Co., Ltd.
4	13850	Gongkong (Beijing) Information Technology Co., Ltd.
5	13734	Business-Intelligence of Oriental Nations Co., Ltd.
6	3969	Beijing Shenzhou Taiyue Intelligent Data Technology Co., Ltd.
7	23142	Beijing Zhiyun Qidian Technology Co., Ltd.
8	15798	Beijing Libiao Xinzhi Technology Co., Ltd.
9	9911	Z-Park Software Park Development Co., Ltd.
10	11832	Wisdom Craftsman (Beijing) Science and Technology Co., Ltd.
11	3943	Beijing Baidu Network Technology Co., Ltd.
12	18465	China Software & Technology Services Co., Ltd.
13	5085	Beijing Guoxin Tianchen Information Security Technology Co., Ltd.
14	5493	YIYANG Security Technology Co., Ltd.
15	5677	Beijing Chen’an Technology Co., Ltd.
16	21925	ChinaSoft Information System Engineering Co., Ltd.
17	13402	Beijing iQIYI Science and Technology Co., Ltd.
18	2325	Jingshuo Century Science and Technology (Beijing) Co., Ltd.
19	9877	Beijing Donghua Software Co., Ltd.
20	13602	Beijing Huasheng Tiancheng Technology Co., Ltd.

**Table 4:** The 20 high-tech enterprises with the highest betweenness centrality under the ENMON model.

Ranking	Company No.	Company Name
1	3943	Beijing Baidu Network Technology Co., Ltd.
2	10479	Beijing Sina Internet Information Services Co., Ltd.
3	13850	Gongkong (Beijing) Information Technology Co., Ltd.
4	11352	Economic Century Medical Network Technology (Beijing) Co., Ltd.
5	10491	Huilan Technology Co., Ltd.
6	13402	Beijing iQIYI Science and Technology Co., Ltd.
7	17662	Beijing Smart Housekeeper Technology Co., Ltd.
8	12971	Beijing Qihoo Technology Co., Ltd.
9	9601	Tongfang Health Technology Co., Ltd.
10	17455	Zhiqu Life Community Service (Beijing) Co., Ltd.
11	20040	Beijing Internetware Co., Ltd.
12	14031	Huadi Computer Software Co., Ltd.
13	12503	Beijing Capipad Communication Equipment Co., Ltd.
14	5600	Beijing Sogou Information Service Co., Ltd.
15	23142	Beijing Zhiyun Qidian Technology Co., Ltd.
16	14151	Beijing Unisound Information Technology Co., Ltd.
17	19084	Beijing Esafenet S & T Co., Ltd.
18	10241	Baidu Online Network Technology (Beijing) Co., Ltd.
19	13734	Business-Intelligence of Oriental Nations Co., Ltd.
20	12556	ThunderSoft Co., Ltd.

## Data Availability

We signed a confidentiality agreement with the transportation company that provided us with the data used in this work. Hence the data will not be shared.
